# A Handsome Face

**Published:** 1872-09

**Authors:** 


### A HANDSOME FACE

Is an attainable possession, for it is the mind that makes it, and keeps it handsome till threescore and after. It is mental activity, it is a busy life in stirring scenes, in heavy responsibilities, constantly calling out all the latent forces; these are they which give a fire to the eye, a firmness to the lip, and a clear, sharp outline to the features of the face; on the other hand, those who are born handsome soon grow dull and flat and flabby, and jejune in face and form and mind and heart, when they give themselves up to idleness and inactivity and animal indulgences. To get handsome and remain handsome, be busy in the execution of high resolves.

### NOTICE.

PLEASE send all moneys, subscriptions, and inquiries to HURD & HOUGHTON, 13 Astor Place, New York city. — ED.

